# Genetic diversity and antifungal susceptibility of *Candida albicans* isolates from Iranian HIV-infected patients with oral candidiasis

**DOI:** 10.1186/s13104-021-05498-8

**Published:** 2021-03-10

**Authors:** Iradj Ashrafi Tamai, Babak Pakbin, Bahar Nayeri Fasaei

**Affiliations:** Department of Microbiology and Immunology, Faculty of Veterinary Medicine, University of Tehran, P.O. Box: 14155-6453, Tehran, Iran

**Keywords:** *Candida albicans*, HIV^+^ patients, Genetic diversity, RAPD-PCR, Antifungal susceptibility

## Abstract

**Objective:**

The objectives of this study were to investigate the antifungal susceptibility and genetic diversity of *Candida albicans* isolated from HIV^+^ patients with oropharyngeal candidiasis. A total of 50 *C. albicans* isolates were cultured on Sabouraud glucose agar containing chloramophenicol. The antifungal susceptibility of the isolates against fluconazole, clotrimazole, nystatin, amphotericin B, ketoconazole and flucytosine was assessed using disc diffusion method. The genetic diversity of *C. albicans* isolates was determined using random amplified polymorphic DNA marker.

**Results:**

The inhibition zones ranged from 4 ± 1.8 to 40 ± 3.8 mm for fluconazole, 7 ± 1.0 to 37 ± 1.8 mm for ketoconazole, 14 ± 0.8 to24 ± 0.8 mm for amphotericin B, 25 ± 0.0 to 33 ± 0.0 mm for nystatin and 7 ± 4.2 to 40 ± 0.0 mm for clotrimazole. At 90% similarity, three distinct groups were observed. The smallest cluster composed of 3 isolates, whereas the largest one composed of 17 isolates. 32% (16/50), 28% (14/50) and 14% (7/50) were resistant to fluconazole, ketoconazole and clotrimazole, respectively.

## Introduction

*Candida albicans* (*C. albicans*) has been known as one of the most important clinical pathogen [1]. Oropharyngeal candidiasis (OPC) is frequently because of the increase of predisposing factors such as hematologic malignancy, acquired immunodeficiency syndrome (AIDS), Nezelof syndrome, zinc and iron deficiency. OPC, as the independent predictor of immunodeficiency in AIDS patients, increases the mortality and morbidity among these patients; consequently, it requires prompt therapy and precise diagnosis [2]*. C. albicans*, only species recovered from up to 70% of HIV-infected individuals, is one of the most common cause of mucosal yeast infection in human [3]. The prevalence of oral *Candida* infections in patients with HIV has recently been decreased. Two factors described this phenomenon; first, overuse of antifungal agents, particulary azole antibiotics; second, the introduction of highly active antiretroviral therapy resulted in a significant decrease in the incidence of opportunistic diseases and the mortality of AIDS [4]. Since azoles, particularly fluconazule, have been used for prophylaxis or treatment of AIDS, resistance to this antifungal agent is concerning [5]. Resistance can be acheived by an alteration of the target enzyme, the cytochrome P-450 lanosterol 14 α-demethylase, mediated by ERG11 gene, or the failure of azole antifungal to accumulate inside the cell as a result of enhanced drug efflux, mediated by MDR and CDR genes [6].

Recent progresses in molecular typing methods have been generated by several techniques based on PCR, including multi-locus enzyme electrophoresis (MLEE), restriction enzyme analysis (REA), randomly amplified polymorphic DNA (RAPD) analysis and karyotype analysis for evaluation of genetic diversity and molecular epidemiology [7]. In recent years, RAPD has been increasingly employed as a molecular method for investigation of population genetics and genotyping of different microorganisms [8]. The genetic diversity of various *Candida* species and the correlation between the antifungal susceptibility and gene diversity have previously been studied. The RAPD assay relies on the use of arbitrary primers that are annealed to DNA. This technique has become one of the most commonly used methods for DNA fingerprinting of clinical *Candida* species [9]. The purposes of the present study were to determine the antifungal susceptibility and the genetic diversity of *C. albicans* isolates collected from oral cavity of Iranian HIV^+^ patients with OPC.

## Main text

### Microorganisms

This study was performed on 50 *C. albicans* isolates collected from oral cavity of HIV^+^ patients, from October to November 2011, at the AIDS Research and Training Center of Imam Khomeini Hospital, Tehran, Iran. HIV-infected Patients, previously diagnosed and confirmed with ELISA test, with the age between 15 and 65 years old and including both genders, were enrolled for the study and referred to the hospital to receive medical advice and health care. They had not any systemic diseases and did not receive any antifungal therapy during the last 3 months; however, they were under treatment for HIV. All confirmed HIV-infected patients with oropharyngeal lesions were included in the study. The most likely potential confounding factors were having systemic diseases and receiving antifungal treatment which were considered to minimize the effects of possible confounding factors. For the collection of speciemens, oral swabs were collected from the lesions with a sterile swab. We used a wet mount with 10% KOH preparation and Giemsa stain for microscopic examination of pseudo-hyphae and yeast cells. All swab samples were cultured on Sabouraud glucose agar (SGA; 20 g/l glucose, 10 g/l peptone, 20 g/l agar, pH 5.6) containing 0.05% chloramphenicol (Merck Co., Darmstadt, Germany). The cultures were incubated at 37 °C and examined daily for 1 week. Identification of *C. albicans* isolates were performed on the basis of germ tube test, colony color on CHROMagar (Paris, France Company), sugar fermentation, assimilation tests by RAPID yeast plus system (Remel Inc., USA). We employed internal transcribed spacer primer pairs including CALB1 and CALB2. Also, we used *C. albicans* ATCC 90028 as the positive control in this study.

### Antifungal susceptibility

Antifungal susceptibility of the isolates were determined using agar disc diffusion assay according to Clinical and Laboratory Standards Institute (CLSI) document M44-A2 [10]. Six standard antifungal discs including fluconazole (25 µg/disc), clotrimazole (10 µg/disc), nystatin (50 µg/disc), amphotericin B (20 µg/disc), ketoconazole (10 µg/disc) and flucytosine (1 µg/disc) were obtained from Oxoid (Hampshire, UK) [11]. Briefly, a suspension of *C. albicans* (10^6^ cell/ml) was spread onto Muller-Hinton agar (Oxoid, Hampshire, UK) plates containing 2% glucose and 0.5 μg/ml methylene blue dye. Antifungal discs were placed on the inoculated plates. These plates were incubated 48 h at 37 °C. The diameter of the inhibition zones was measured in millimeter [12].

### DNA extraction

All samples, which were not treated with the antifungal drugs, were cultured on SGA at 37 °C for 48 h. Genomic DNA of yeast cells was extracted as previously described [13] and purified using a commercial DNA purification kit (Ultraclean Microbial DNA Isolation kit, MO BIO, USA) according to manufacturer`s structure. DNA concentration and purity were determined based on optical density at 260 nm and ratio OD 260/280 nm, respectively.

### PCR assay and RAPD

PCR analysis was performed with the oligonucleotide primers CALB1: TTTATCAACTTGTCACACCAGA and CALB2: ATCCCGCCTTACCACTACCG (The GenBank accession number refers to primers L47111, L28817) [14]. Amplification reactions were done in a final volume of 25 µl containing 2.5 µl of reaction buffer (10×), 1.5 mM Mgcl_2_ (50 mM), 0.2 mM dNTP (10 mM), 0.5 µM (each) primers, 0.5 U of Taq DNA polymerase, 2 µl of genomic DNA template and deionized sterilized water to the final volume. Amplification was carried out using a Techne Tc-512 thermo cycler (Techne, UK). Initial denaturation was at 96 °C for 5 min, as follows: 35 cycles of 30 s for denaturation at 94 °C, 30 s for annealing at 55 °C and 30 s primer extension at 72 °C, followed by a terminal extension at 72 °C for 15 min. The PCR products were electrophoresed on 1.5% agarose gel for 1 h at 80 v and stained with ethidium bromide (2 µg/ml). RAPD-PCR was performed with RSD12: 5′-GGTCCGTGTTTCAAGACG-3′ primer [15]. Each reaction mixture contained 2.5 μl of reaction buffer (10×), 2.5 mM MgCl_2_, 200 mM dNTPs mix, 1.25 μM of primer RSD12, 1U of Taq DNA polymerase and 100–400 ng of DNA template in final reaction volume of 25 μl. PCR amplification program for RSD12 primer involved 1 cycle at 95 °C for 5 min, then 40 cycles as follows: 30 s for denaturation at 94 °C, 2 min for annealing at 57 °C and a final extension step 72 °C for 2 min in a Techne TC-512 thermocycler (Techne, Cambridge, UK). The PCR products were analysed by electrophoresis on 1.5% agarose gel at 70 v for 80 min in TBE buffer (1×), stained in a 0.5 mg/ml ethidium bromide solution for 15 min and photographed by CCD Video Camera. The patterns of DNA banding were analysed employing GelCompar software ver. 6 (Applied Math, Belgium).

### Statistical analysis

The chi-square and t-test using SPSS software version 12.0 (SPSS Inc., Chicago, IL, USA) were performed to statistical analysis. A phenogram was constructed by the Unweighted Pair Group Method with Arithmetic Mean (UPGMA) after determination of association coefficients using the simple matching method.

### Results

The antifungal susceptibility and genetic diversity of 50 clinical *C. albicans* isolates were evaluated by disc diffusion method and RAPD analysis, respectively. The antifungal susceptibilities of *C. albicans* isolated from oral cavity of Iranian HIV^+^ patients are shown in Tables 1 and 2. Results of susceptibility to antifungal drugs were as follows: fluconazole: 29 isolates (58%) susceptible, 5 isolates (10%) susceptible-dose dependent or intermediate and 16 isolates (32%) resistant; ketoconazole: 31 isolates (62%) susceptible, 5 isolates (10%) susceptible-dose dependent or intermediate and 14 isolates (28%) resistant; amphotericin B: 48 isolates (96%) susceptible, 2 isolates (4%) susceptible-dose dependent or intermediate; clotrimazole: 35 isolates (70%) susceptible, 8 isolates (16%) susceptible-dose dependent or intermediate and 7 isolates (14%) resistant; flucytosine: 50 isolates (100%) resistant and nystatin: 50 isolates (100%) susceptible.

**Table 1:** Antifungal susceptibility of oral *Candida albicans* isolates from HIV-infected patients

Antifungal agents	Drug concentration	Resistance (%)	Intermediate (%)	Susceptible (%)
Fluconazole (FCN)	25 μg	32	10	58
Clotrimazole (CTM)	10 μg	14	16	70
Nystatin (NY)	100 units	0	0	100
Amphotericin B (AMB)	20 μg	0	4	96
Ketoconazole (KCA)	10 μg	28	10	62
Flucytosine (FY)	1 μg	100	0	0

**Table 2:** Antifungal susceptibility of the standard antifungal agents against *Candida albicans* isolates (mean ± standard deviation, millimeter)

Genotype	Fluconazole	Ketoconazole	Amphotericin B	Nystatin	Clotrimazole
C1	9 ± 1.2	8 ± 1.8	20 ± 1.8	26 ± O.2	11 ± 1.4
C2	11 ± 0.8	11 ± 2.4	17 ± 2.0	25 ± 0.0	19 ± 2.2
C3	18 ± 0.0	25 ± 0.8	20 ± 3.2	30 ± 0.0	36 ± 0.8
C4	30 ± 2.2	32 ± 2.0	14 ± 0.8	27 ± 0.0	32 ± 0.8
C5	35 ± 2.6	30 ± 0.4	18 ± 0.0	26 ± 0.0	33 ± 1.0
C6	9 ± 1.4	7 ± 1.0	17 ± 1.0	25 ± 0.8	10 ± 2.2
C7	12 ± 1.8	28 ± 2.2	17 ± 2.8	25 ± 0.4	18 ± 1.4
C8	33 ± 1.2	31 ± 3.6	18 ± 1.4	25 ± 0.0	33 ± 4.2
C9	29 ± 2.4	32 ± 0.8	18 ± 4.2	25 ± 0.0	25 ± 2.6
C10	12 ± 1.4	28 ± 0.0	18 ± 2.0	27 ± 0.0	17 ± 1.8
C11	5 ± 0.8	11 ± 0.2	23 ± 1.8	25 ± 0.4	9 ± 2.6
C12	37 ± 1.8	31 ± 1.2	17 ± 1.4	25 ± 0.0	30 ± 0.8
C13	9 ± 0.8	28 ± 0.6	16 ± 2.2	28 ± 0.0	8 ± 3.4
C14	35 ± 1.0	33 ± 0.2	18 ± 0.0	25 ± 0.0	35 ± 0.8
C15	8 ± 2.6	10 ± 0.8	16 ± 3.3	26 ± 0.8	12 ± 2.4
C16	18 ± 1.0	28 ± 0.0	17 ± 1.0	25 ± 0.6	28 ± 0.0
C17	35 ± 3.2	34 ± 0.0	15 ± 5.0	25 ± 0.0	26 ± 0.8
C18	39 ± 0.8	35 ± 2.8	16 ± 0.8	26 ± 1.2	25 ± 0.6
C19	6 ± 0.0	15 ± 0.0	15 ± 2.0	25 ± 0.0	9 ± 3.8
C20	40 ± 3.8	35 ± 0.8	16 ± 3.2	26 ± 0.0	30 ± 2.6
C21	32 ± 2.0	32 ± 2.4	15 ± 1.0	25 ± 0.0	28 ± 6.2
C22	4 ± 1.8	7 ± 3.4	16 ± 0.8	26 ± 0.0	17 ± 0.8
C23	17 ± 0.0	25 ± 0.8	16 ± 3.4	25 ± 0.8	25 ± 1.6
C24	29 ± 2.2	21 ± 2.8	15 ± 3.2	25 ± 0.8	30 ± 2.4
C25	18 ± 1.0	25 ± 3.2	15 ± 1.8	27 ± 0.0	40 ± 0.0
C26	35 ± 0.8	32 ± 2.0	15 ± 3.6	27 ± 0.0	23 ± 3.6
C27	5 ± 3.8	8 ± 4.8	16 ± 1.8	25 ± 1.2	7 ± 4.2
C28	31 ± 0.6	35 ± 0.8	17 ± 0.8	27 ± 0.8	30 ± 1.6
C29	30 ± 1.9	31 ± 2.0	16 ± 0.0	25 ± 0.0	25 ± 2.6
C30	5 ± 4.0	29 ± 1.8	18 ± 1.0	28 ± 1.6	13 ± 0.8
C31	10 ± 1.2	17 ± 1.6	16 ± 4.0	25 ± 0.0	25 ± 1.2
C32	28 ± 3.0	28 ± 4.2	16 ± 2.0	25 ± 0.0	27 ± 3.8
C33	21 ± 2.8	14 ± 2.0	17 ± 1.4	25 ± 0.0	24 ± 2.2
C34	18 ± 0.0	15 ± 0.0	16 ± 0.6	23 ± 0.0	25 ± 2.8
C35	27 ± 1.4	31 ± 0.8	16 ± 0.0	26 ± 0.8	35 ± 0.8
C36	7 ± 1.6	9 ± 0.8	15 ± 3.4	26 ± 0.0	17 ± 4.2
C37	33 ± 4.8	37 ± 1.8	17 ± 2.4	26 ± 0.0	26 ± 3.2
C38	4 ± 3.6	10 ± 1.0	18 ± 2.8	28 ± 0.0	19 ± 1.4
C39	31 ± 0.8	30 ± 2.8	18 ± 1.4	28 ± 0.0	32 ± 4.2
C40	29 ± 2.4	30 ± 0.0	16 ± 4.2	26 ± 1.4	35 ± 0.8
C41	34 ± 1.8	31 ± 0.6	17 ± 2.4	26 ± 1.2	29 ± 3.2
C42	32 ± 2.8	34 ± 2.0	20 ± 1.0	29 ± 0.0	31 ± 2.8
C43	37 ± 2.4	30 ± 1.4	18 ± 3.2	33 ± 0.0	29 ± 1.0
C44	29 ± 3.8	31 ± 4.0	22 ± 2.0	31 ± 0.0	29 ± 0.0
C45	37 ± 2.0	29 ± 3.2	24 ± 0.8	30 ± 0.2	33 ± 0.8
C46	30 ± 0.0	21 ± 1.6	17 ± 2.8	27 ± 0.0	31 ± 2.0
C47	8 ± 2.8	9 ± 2.4	14 ± 6.0	31 ± 1.4	8 ± 1.6
C48	31 ± 0.8	33 ± 1.8	18 ± 3.2	25 ± 1.0	30 ± 0.8
C49	29 ± 3.4	30 ± 0.8	17 ± 2.6	30 ± 0.0	31 ± 2.0
C50	34 ± 2.2	33 ± 3.2	22 ± 1.8	31 ± 0.0	30 ± 0.0
ATCC	30 ± 1.8	30 ± 2.8	19 ± 0.8	27 ± 0.0	35 ± 0.8

The inhibition zone diameters ranged from 4 ± 1.8 to 40 ± 3.8 mm (mean value 23.0 ± 11.7 mm) for fluconazole; from 7 ± 1.0 to 37 ± 1.8 mm (mean value 24.8 ± 9.4 mm) for ketoconazole; from 14 ± 08 to 24 ± 08 mm (mean value 17.2 ± 2.1 mm) for amphotericin B; from 7 ± 4.2 to 40.0 mm (mean value 24.8 ± 8.7 mm) for clotrimazole; and from 25 ± 0.0 to 33 ± 0.0 mm (mean value 26.6 ± 2.0 mm) for nystatin. All isolates were resistant to flucytosine (no inhibition zone was observed).

**Fig. 1:**
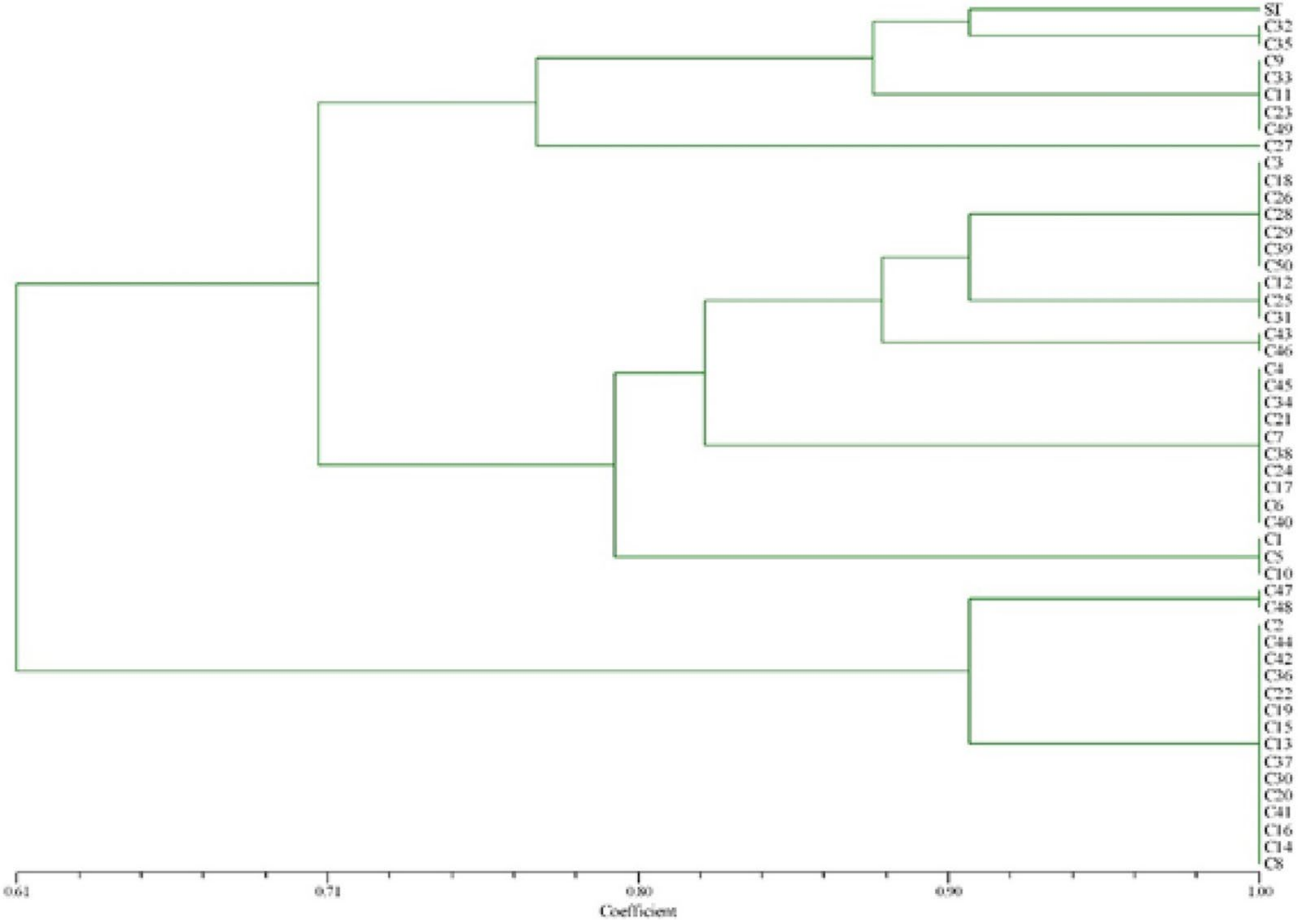
Dendrogram based on RAPD-PCR data for *Candida albicans* isolates from oral cavity of Iranian HIV^ +^ patients. Similarity coefficients ≥ 90%; containing from 3 to 17 isolates each genotype; encompassed 30 isolates and 13 genotypic particular strains. The first cluster (the smallest one) composed of ramifications a (ATCC strain) and a′ (C32 and C35 isolates); the second cluster, ramifications b and b′; and the third cluster (the largest one) composed of ramifications c and c′

*Candida albicans* isolates yielded RAPD profiles with one strong band, with molecular size of 273 bp. Regarding the RAPD-PCR profiles (Fig. 1) and similarity coefficients ≥ 90%; genotypes, containing from 3 to 17 isolates each, encompassed 30 (58.82%) isolates and 13 (25.49%) genotypic particular strains. The first cluster, which was the smallest one, composed of ramifications a (ATCC strain) and a^′^ (C32 and C35 isolates), the second cluster composed of ramifications b and b^′^, and the third cluster (the largest one) composed of ramifications c and c^′^, as shown in Fig. 1.

### Discussion

Genetic fingerprinting of *C. albicans* isolates has widely been studied by several researchers [16]; however, there is limited information about the correlation between the antifungal susceptibility and genetic diversity among *C. albicans* isolated from HIV-infected individuals [17]. Genetic diversity may occur in the population of *C. albicans*, revealed by RAPD profiles, because of developed resistance to fluconazole which has previously been described; and we have also observed that at the present study [18]. We used a specific primer pair (CALB1 and CALB2) to amplify a 273 bp region located on 5.8 rRNA gene and identify the *C. albicans* isolates. These results are in line with that of Sharifzadeh et al. [19]. The results of genotyping with RSD12 primer indicated different genetic profiles among *C. albicans* isolates with different antifungal susceptibility patterns. Hamzehee et al. carried out RAPD-PCR for genotyping of *C. albicans* isolates and they found 46 genotypes including 11 clusters with similarity coefficient of ≥ 80% [9]. Ethtedar-Nejad et al. used PCR-HRM for genotyping of *C. albicans* isolates and they found this method reliable for accurate identification of *Candida* species in clinical samples; however, this method is more expensive than RAPD method for genotyping in clincal settings [20]. Sun et al. used RSD6 primer and assessed genetic diversity of *C. albicans* isolated from root canal infection. They found 31 genotypes among 37 isolates [21]. RSD10 and RSD12 primers also were used to determine the clonal variability of 443 *C. albicans* isolates collected from 16 HIV-infected individuals. These isolates formed clusters comprising 2 or more strains at similarity coefficient of ≥ 80% [22]. Our results were also in agreement with those of previous studies.

Fluconazole, despite using drug potency up to 25 mg per disc, showed poor activity against the isolates tested. There are also many studies indicating that fluconazole had less activity against *Candida* species [23]. Fluconazole resistance, in spite of using drug potency up to 25 µg per disc, was 32%. Higher rates of resistance which we observed were not in accordance with those observed in United Kingdom, Brazil, Mexico and other studies which showed lower levels of antifungal drugs resistance [24]. The reason for higher fluconazole resistance could be explained by the fact that azoles, especially fluconazole, have been used for prophylaxis or incomplete treatment of oral candidiasis in HIV-infected patients, therefore, resistance to fluconazole is common during AIDS-related complex [25].

### Significance of the study

*C. albicans* has been known as one of the most frequent yeasts species associated with infections in immunocompromised patients, such as HIV-infected individuals [26]. The findings of the current study revealed genetic diversity and correlation between antifungal susceptibility profiles and the genotype groups of *C. albicans* isolated from oral cavity of HIV^+^ patients. We also found that amphotericin B and nystatin were the most effective antifungal drugs and fluconazole showed the poorest activity against *C. albicans*.

## Limitations

- Despite all efforts, 50 isolates is not efficient to study and evaluate the genetic relatedness and anti-fungal susceptibility of *C. albicans* isolated from HIV-infected individuals.
- RAPD is a popular genotyping approach; however, it is not precise enough because of lack of reproducibility. Other genotyping assays are suggested to be implemented.

## Data Availability

All data supporting the results and conclusion of this paper are included within the article.

## Abbreviations

OPC: Oropharyngeal candidiasis
AIDS: Acquired immunodeficiency syndrome
MDR: Multidrug resistant
RAPD: Randomly amplified polymorphic DNA
HIV: Human immunodeficiency virus
